# A Tunable Plasmonic Refractive Index Sensor with Nanoring-Strip Graphene Arrays

**DOI:** 10.3390/s18124489

**Published:** 2018-12-18

**Authors:** Chunlian Cen, Hang Lin, Jing Huang, Cuiping Liang, Xifang Chen, Yongjian Tang, Zao Yi, Xin Ye, Jiangwei Liu, Yougen Yi, Shuyuan Xiao

**Affiliations:** 1 Joint Laboratory for Extreme Conditions Matter Properties, Southwest University of Science and Technology, Mianyang 621010, China; cenchunlian@mails.swust.edu.cn (C.C.); lh9711100@yeah.net (H.L.); h2311320325@yeah.net (J.H.); lcp144@yeah.net (C.L.); chenxifang1988@yeah.net (X.C.); Tangyongjian2000@sina.com (Y.T.); 2 Research Center of Laser Fusion, China Academy of Engineering Physics, Mianyang 621010, China; 3 Sichuan Civil-Military Integration Institute, Mianyang 621010, China; 4 School of Energy Science and Engineering, Central South University, Changsha 410083, China; jiangweiliu@csu.edu.cn; 5 College of Physics and Electronics, Central South University, Changsha 410083, China; 6 Institute for Advanced Study, Nanchang University, Nanchang 330031, China; syxiao@hust.edu.cn

**Keywords:** surface plasmon resonance, refractive sensing, graphene

## Abstract

In the present study, we design a tunable plasmonic refractive index sensor with nanoring-strip graphene arrays. The calculations prove that the nanoring-strip have two transmission dips. By changing the strip length *L* of the present structure, we find that the nanoring-strip graphene arrays have a wide range of resonances (resonance wavelength increases from 17.73 μm to 28.15 μm). When changing the sensing medium refractive index *n_med_*, the sensitivity of mode A and B can reach 2.97 μm/RIU and 5.20 μm/RIU. By changing the doping level *n_g_*, we notice that the transmission characteristics can be tuned flexibly. Finally, the proposed sensor also shows good angle tolerance for both transverse magnetic (TM) and transverse electric (TE) polarizations. The proposed nanoring-strip graphene arrays along with the numerical results could open a new avenue to realize various tunable plasmon devices and have a great application prospect in biosensing, detection, and imaging.

## 1. Introduction

Surface plasmon resonance (SPR) on the metal/dielectric interface at the sub-wavelength scale provides an excellent platform for a variety of optoelectronic applications [1,2]. SPR properties depend on the nanostructure of cell plasmon geometry, size, composition and optical polarization [3,4,5,6]. This particular feature is extensively used in the field of biological or chemical sensing [7]. According to the electronics and photonics properties, a number of applications have been reported, including ultra-fast transistor photodetectors [8,9,10], light emitters [11,12], optical modulators [13,14,15], optoelectronic devices [16] and transparent solar cells [17].

Graphene, a monolayer of carbon atoms, arranged in a plane with a honeycomb lattice, due to its unique optical and electronic properties, it has stimulated research interest in photonics and optoelectronics [18]. More interestingly, the electric field, magnetic field, and chemical doping can effectively adjust the surface conductivity of graphene [19,20]. Due to its unique electrical and optical properties [21,22], it has been widely used in the fields of transparent electrode [23,24,25], light modulator [26,27], and photoelectric detector [28,29,30], and has great potential for development in these fields.

In the present study, we design a tunable plasmonic refractive index sensor with nanoring-strip graphene arrays. The transmission response relying on geometrical sizes of the nanostructure and the sensing medium refractive index are extensively studied by the finite-difference time-domain (FDTD) method. By changing the strip length *L* of the present structure, we find that the nanoring-strip graphene arrays have a wide range of resonances (resonance wavelength increases from 17.73 μm to 28.15 μm). When changing the sensing medium refractive index *n_med_*, the sensitivity of mode A and B can reach 2.97 μm/RIU and 5.20 μm/RIU. Such a high sensitivity will have great prospects in terms of biosensing and detection. We finally investigate the oblique incidence of incident electromagnetic waves and the results show that the resonance is angle-insensitive. The resonance is confined to the electromagnetic field at the edge of the nanoring-strip. In addition, the nanoring-strip with different doping level for electrically tunable spectral imaging provides interesting applications. The proposed sensor structure can be used in biosensing, detection, and imaging.

## 2. Materials and Methods

**Figure 1:**
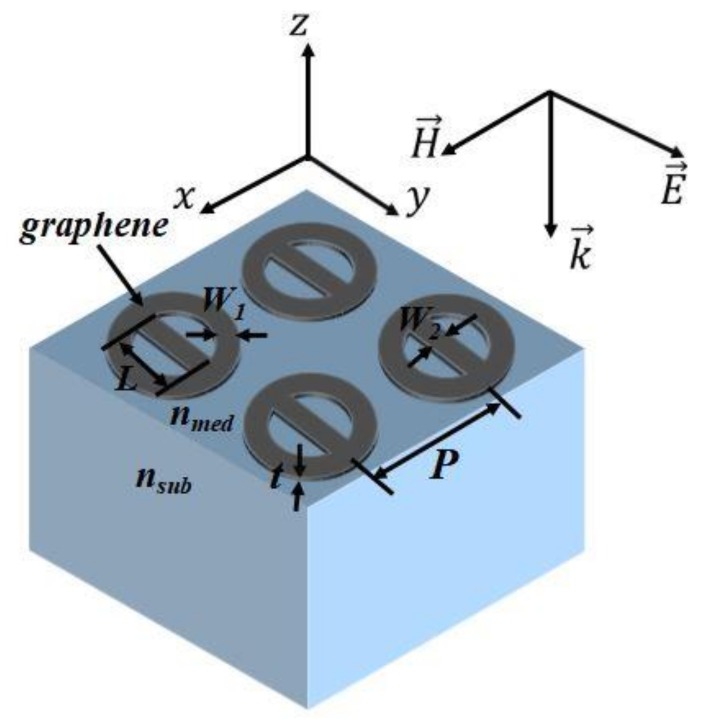
The schematic design of the geometry is as follows: nanoring-strip graphene arrays with period *P* = 300 nm, strip length *L* = 180 nm, nanoring width *W*_1_ = 30 nm, strip width *W*_2_ = 30 nm, and graphene thickness *t* = 1 nm. The arrays arranged in a substrate (*n_sub_*) and a sensing medium (*n_med_*).

In Figure 1, we present a simple nanoring-strip structure. It includes of a nanoring-strip graphene arrays arranged in a substrate (*n_sub_*) and a sensing medium (*n_med_*). We assume that the substrate is semi-infinite. *P* = 300 nm is the fixed period of the arrays. The width of the nanoring is *W_1_* = 30 nm and the thickness of graphene is *t* = 1 nm. The strip width is *W_2_* = 30 nm and the length is *L* = 180 nm. 

Graphene surface conductivity can be obtained through the Kubo formula [31,32,33], containing the intraband transition contributions and interband. But, in the lower THz frequency range, the interband transition and optical phonon emission contributions are very low, and their effects can be ignored [34,35]. Further, in the calculation process, the temperature (*T*) is set to 300 K and considering the doping level of graphene and the condition *E_F_* >> *K_B_T* can be satisfied. In general, in accordance with the Pauli Exclusion Principle conductive of the graphene surface can be approximated as an intraband Drude model expression:

(1) $$\sigma (\omega )=\frac{e^{2}E_{F}}{\pi \hbar^{2}}\frac{i}{\omega +i/\tau },$$

Here, the ω is angular frequency of the incident wave, the *e* is the charge of electron, the *ћ* = *h*/2π is the reduced Planck constant and the *τ* is the relaxation time. Where, the *τ* and *E_F_* can be written as

(2) $$\tau =\mu \hbar \sqrt{\pi {|n}_{g}|}/ev_{F},$$

and

(3) $$E_{F}=\hbar V_{F}\sqrt{\pi |n_{g}|},$$

*n_g_*, *ν_F_* = 106 m/s and *µ* ≈ 10,000 cm^2^/(V·s) are the graphene doping level, the velocity of graphene Fermi and the measured dc mobility, respectively [18].

In this research, we calculate the graphene nanoring-strip spectral responses using the FDTD method. We use FDTD method with the software FDTD Solutions to calculate transmission spectra and electric field distributions [36,37]. In the whole calculation process, the periodic boundary conditions in the *y* and *x* directions are adopted, respectively. Then using the perfectly matched layer (PML) boundary conditions employed in the incident light wave propagating along the *z*-direction. In the entire analog computing system, light travels along the negative *z*-axis, illuminating the entire graphene array with a polarization direction of the *y*-axis. We use the plane wave to perform the corresponding simulation calculation.

## 3. Results and Discussion

**Figure 2:**
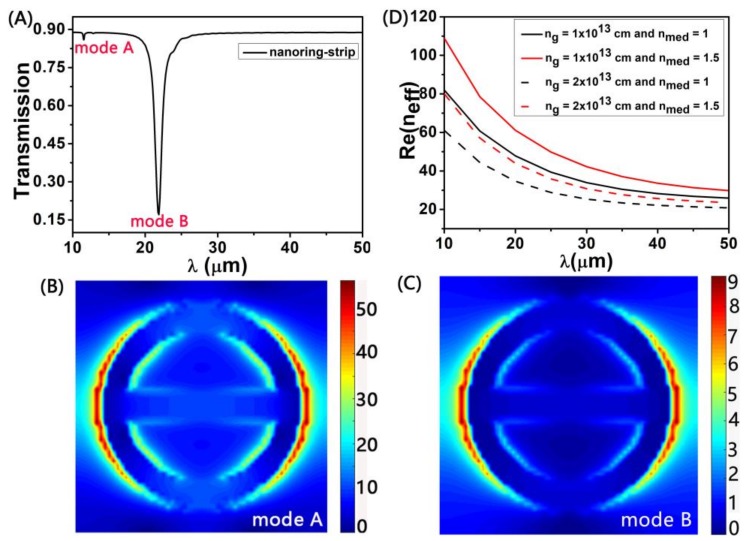
(**A**) The transmission spectra of the nanoring-strip (strip length *L* = 180 nm, nanoring width *W_1_* = 30 nm, and strip width *W_2_* = 30 nm). (**B**) and (**C**) The electric field distribution of nanoring-strip structure in shorter wavelength (mode A) and longer wavelength (mode B), respectively. (**D**) Calculated effective refractive indices of different the *n_g_* and the sensing medium refractive index *n_med_*.

We first investigate the transmission spectra of nanoring-strip (strip length *L* = 180 nm, nanoring width *W_1_* = 30 nm, strip width *W_2_* = 30 nm, and graphene thickness *t* = 1 nm, respectively). As shown in Figure 2A, we can obtain these transmission spectra of the nanoring-strip. In short wavelength region, we find that there is a small transmission dip. As shown in Figure 2B, we find that the electric field of the nanoring-strip is mainly distributed on the nanoring arm on both sides of the edge of the inner and outer at the mode A, which makes it has a smaller transmission tip. We can find that the nanoring-strip has an obvious transmission dip (in long wavelength region). This phenomenon can be understood by the electric field distribution diagram, in Figure 2C. We find that the electric field of the nanoring-strip is mainly distributed on the nanoring arm edge of the structure and has stronger field enhancement. In both of Figure 2B,C, because the *x*-polarization of incident light is symmetrical with the *y*-axis, the *x*–*y* plane the electric field distribution (|*Ez*|) at resonance exhibits the characteristics of an electric dipole. The transverse electric dipole resonance enhances local resonance, which effectively captures light energy [38,39].

In theory, the effective wavelength of the above dipole resonance is approximately equal to the perimeter of our proposed nanoring structure [31,34]:

(4) $$\lambda_{eff}=2\pi R.$$

Therefore, the transmission dip wavelength in the graphene nanoring resonator can be indicated as:

(5) $$\lambda_{res}=\lambda_{eff}n_{eff}=2\pi Rn_{eff}.$$

Here, *n_eff_* is the effective refractive index of graphene nanoring waveguide. In Figure 2D, we calculate effective refractive index of different the *n_g_* and the *n_med_* for the same width (*W* = 30 nm) of graphene ribbon waveguide. Thus we can get neff a function of *n_g_* and *n_med_*. From the Equation (5), we know that by changing the *n_med_*, the transmission dip wavelength *λ_res_* can be shifted, which is the principle nanoring structure for sensing applications. In addition, the *n_g_* variation value can also change the *λ_res_*, and the influence of *n_g_* on *λ_res_* is greater than that of the *n_med_* on a *λ_res_*. Therefore, the nanoring-strip graphene arrays can realize active tuning of the detection region based on the sensor nanostructure by changing the *n_g_*.

**Figure 3:**
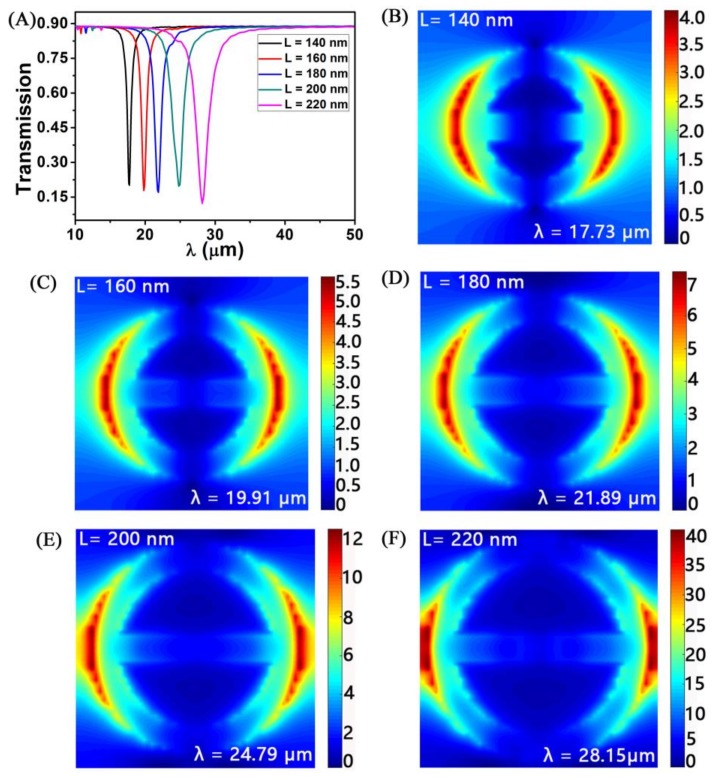
(**A**) The transmission spectra of graphene with different strip length (*L*). Structural parameters: *W_1_* = *W_2_* = 30 nm, *P* = 300 nm, *n_g_* = 3 × 10^13^ cm^−2^, and *n_med_* = 1.0. *L* = 140 nm, 160 nm, 180 nm, 200 nm, and 220 nm the corresponding electric field distribution is labeled (**B**)–(**F**).

We study the geometric structure of the nanoring-strip graphene arrays. Other parameters are unchanged (*W_1_* = *W_2_* = 30 nm, *P* = 300 nm, *n_g_* = 3 × 10^13^ cm^−2^, *t* = 1 nm, and *n_med_* = 1.0). When we change the strip length *L* from 140 nm to 220 nm, the transmission dip red-shift from 17.73 to 28.15 μm, as shown in Figure 3A. As the strip length *L* increases, the spacing between the adjacent nanoring-strip graphene arrays will be reduced, resulting in increased coupling effect between them. According to formula (5), the transmission dip wavelength increases as the raise of *L*. Increasing the coupling will result in a red-shift of transmission dips. When *L* is increased from 140 to 200 nm, the transmission dip remains almost at 0.20. However, when *L* increases from 200 to 240 nm, the transmission dip decreases to 0.12. This physical mechanism is attributed to these electric field distribution maps, and we can see it from Figure 3B. The change of *L* from 140 to 200 nm with an interval of 20 nm. The corresponding electric field distribution is shown in Figure 3B–E. We can observe that their electric fields are mainly at the edge of nanoring and the distribution intensity is consistent. This confirms that the transmission dip is the same as *L* increases from 140 to 200 nm. The electric field distribution at *L* = 220 nm is shown in Figure 3F. We can clearly see that its electric field is mainly distributed at the edge of nanoring, but it is stronger than the other four lengths, resulting in *L* = 220 nm has a stronger transmission dip.

**Figure 4:**
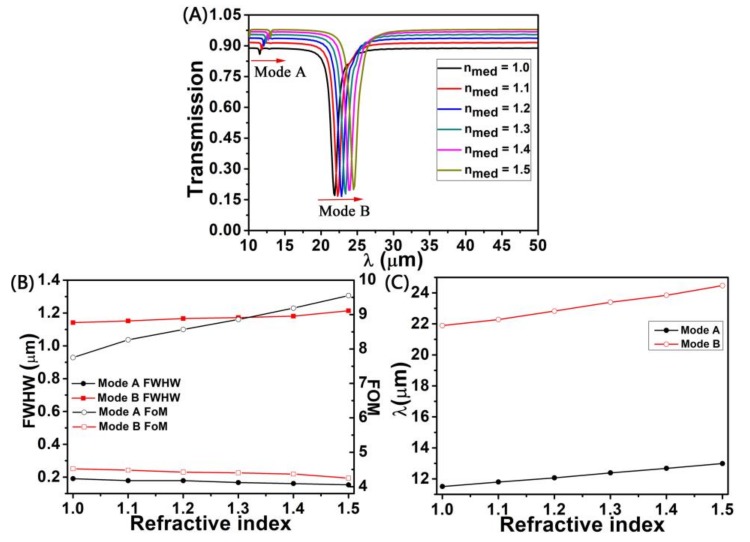
(**A**) The transmission spectra of graphene with different sensing medium refractive index (*n_med_*); (**B**) full width at half maximum (FWHM) and figure of merit (FOM) of mode A and mode B for different the *n_med_*; (**C**) For mode A and mode B, the transmission dip wavelength corresponding to transmission dip as a function of the *n_med_*. Structural parameters: *L* = 180 nm; *P* = 300 nm; *W*_1_ = *W*_2_ = 30 nm; and *n_g_* = 3 × 10^13^ cm^−2^.

We investigate the sensing properties of graphene nanoring-strip system, the transmission spectra of nanoring-strip graphene arrays and plasmon resonance dip at different refractive indices of the surrounding sensing medium (*n_med_*) were simulated by FDTD. It is clear in Figure 4A that the transmission spectra of mode A and mode B have changed significantly in the refractive index of different *n_med_*. Clearly seen in Figure 4A with different the *n_med_*, obvious changes in the transmission spectra of mode A and mode B have taken placed, at the same time, a wide sensing range of the nanoring-strip graphene arrays can be obtained. We also find that there are some changes in the transmittance amplitude and the reason is that the surrounding sensing medium refractive index change will affects the plasmon resonance amplitude. For the sake of quantifying the refractive index of the sensor of the presented performance, we calculate the full width at half maximum (FWHM) and figure of merit (FOM) with different the refractive index for mode A and mode B, in Figure 4B. With regard to mode A and mode B, it is obvious that the shift of the transmission dip wavelength shows linear relationship with the vary of the refractive index, i.e., [40,41]

(6) Δλ=mΔn.

Here, Δλ, m, and Δn are transmission dip wavelength, the transmission dip wavelength shift per refractive index unit (RIU) change and the per unit is RIU and the range of the sensing medium refractive index *n_med_*, respectively. We can get the sensitivity *S* = *m* = Δλ/Δ*n* = əλ/ə*n* by using the above formula [41]. Because the transmission dip wavelength range of the short wavelength is less than the range of the long wavelength, as shown in Figure 4A. The sensitivity of mode A and mode B are 2.97 μm/RIU and 5.20 μm/RIU, respectively. That is, the sensitivity of mode B is higher than the mode A. The FWHM of mode A and mode B both can increase linearly with increasing the value of *n_med_*. That is because the increase of *n_med_*, which can raise the wave vector in the nanoring waveguide and increase the damping of the dipole modes, thereby increases the FWHW of the transmission curve [42]. According to the formula FOM = *m*/FWHW [40], the FOM decreases as the FWHW increases. Namely, the FOM decrease as the refractive index *n_med_* decreases, as shown in Figure 4B.

We also study a plot of variation in transmission dip wavelength with the different the *n_med_*, as shown in Figure 4C. We find that the transmission dip wavelength of mode A and mode B was shifted with different *n_med_*. With the *n_med_* increase, the transmission dip go through a red-shift. That’s because the transmission dip wavelength increases as *n_med_* increases, which are obtained from Equation (3).

**Figure 5:**
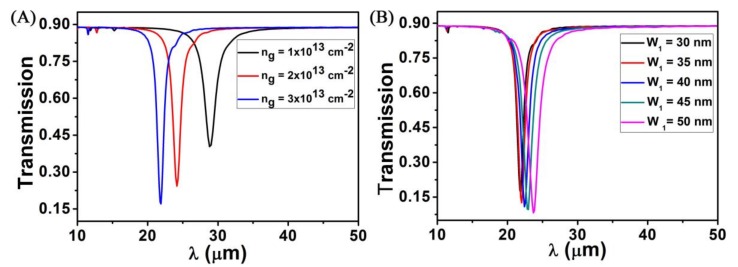
(**A**) Calculated the transmission spectra at different doping levels (*n_g_*); (**B**) Calculated transmission spectra at different ring width (*W*_1_). Other geometry parameters are set to *L* = 180 nm, *W*_2_ = 30 nm, *P* = 300 nm, and *n_med_* = 1.0.

To analyze the tunable properties of the ring structure we have proposed, when *L* = 180 nm, *W*_1_ = *W*_2_ = 30 nm and *P* = 300 nm, we explore the transmission spectra of the structure by changing the *n_g_*, as shown in Figure 5A. We find that as the raise of *n_g_*, graphene nanoring-strip have stronger transmission dip. Therefore, changing the *n_g_* can effectively change the transmission spectra of graphene nanoring-strip, it shows that the structure has a great tunable characteristic. This will apply to the field of sensors. For different *W_1_* values, the *n_g_* and the duty ratio arrays are a constant value. Therefore, in Figure 5B, the corresponding transmission spectra performance almost the same minimum value. In Figure 5B, when the width (*W*_1_) varying from 30 nm to 50 nm, the transmission spectra also have the very high sensitivity, but its transmission dip wavelength change is very small. Through comparing with Figure 3, we find that the strip length of the graphene nanoring-strip has a wider transmission dip wavelength.

**Figure 6:**
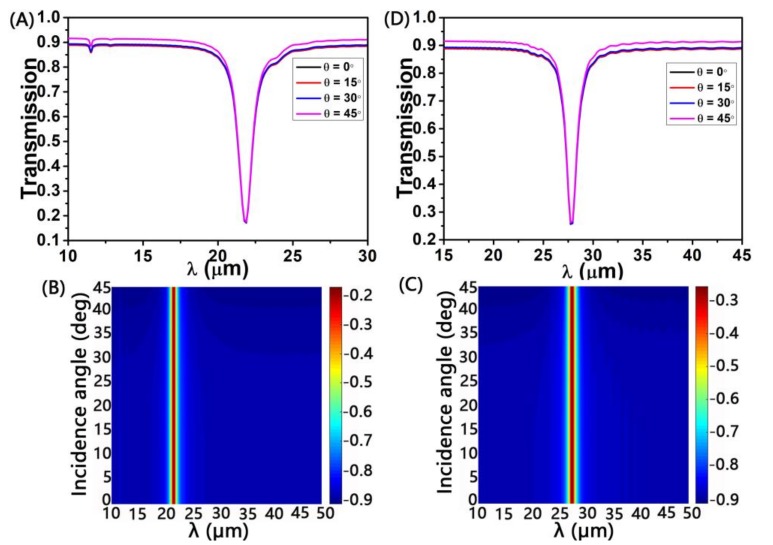
The transmission spectra at different angles of incidence, for TM (**A**) and TE (**B**) polarizations, respectively. The simulated angle dispersions of the transmission in graphene nanoring-strip with the doping level of *n_g_* = 3 × 10^13^ cm^−2^ for (**C**) TM and (**D**) TE.

For TM and TE polarizations, the transmission spectra of the proposed nanostructure at different *θ* were calculated, as shown in Figure 6A,B. For TM polarization, the resonance dip wavelength remains constant at 21.89 μm as the angle of incidence increases. However, for TE polarization, the resonance dip wavelength is maintained at a larger value of 27.74 μm as the angle of incidence increases. In Figure 6A,B, the plasmon resonance dips remain unchanged and the transmission dip over a range of *θ* [0º, 45º] for both TM and TE polarizations. This result shows that for TM and TE polarization, the resonance dip wavelength is insensitive to the angle of incidence. There are two main reasons. Firstly, from Figure 2B,C we can concluded that the electric field of the nanoring-strip is mainly distributed on the inner and outer arms of nanoring. That is, the generation of transmission dips is mainly related to nanoring, and the effect of strip is negligible. Therefore, we can approximate our proposed nanoring-strip structure to a nanoring structure, which is a symmetric structure of nanoring. In addition, the transmission dip here is derived from the strongly localized surface plasmonic resonance. In the periodic conditions, we simulated angle dispersions of the transmission in graphene nanoring-strip with the doping level of *n_g_* = 3 × 10^13^ cm^−2^ for TM and TE polarizations, as shown in Figure 6C,D, respectively. In Figure 6A–D, the plasmon resonance dips at different incident angles are very stable, indicating that the graphene nanoring-strip arrays is insensitive. Consequently, the graphene ring-strip nanoarrays has great prospects in the angle-independent devices.

## 4. Conclusions

In conclusion, a tunable plasmonic refractive index sensor with nanoring-strip graphene arrays has been proposed and theoretically demonstrated. By FDTD method simulation calculation, it is found that the graphene nanoring-strip structure has a high sensitivity to the sensing medium refractive index changes. By changing the strip length *L* of the present structure, we find that the nanoring-strip graphene arrays have a wide range of resonances (resonance wavelength increases from 17.73 to 28.15 μm). When changing the sensing medium refractive index *n_med_*, the sensitivity of mode A and B can reach 2.97 μm/RIU and 5.20 μm/RIU. Such a high sensitivity will have great prospects in terms of biosensing and detection. In addition, the transmission dip resonance wavelength of the proposed nanostructure can be tuned flexibly by changing the doping level *n_g_*. The results also show that the graphene nanoring-strip arrays is insensitive to different polarization modes (TM or TE). From the above results, we conclude that the nanoring-strip graphene arrays have good tunability and high sensitivity, we believe that this sensor has broad application prospects in biosensing, detection, and imaging.
